# Early detection of norovirus outbreak using machine learning methods in South Korea

**DOI:** 10.1371/journal.pone.0277671

**Published:** 2022-11-16

**Authors:** Sieun Lee, Eunhae Cho, Geunsoo Jang, Sangil Kim, Giphil Cho

**Affiliations:** 1 Department of Mathematics, Pusan National University, Busan, Republic of Korea; 2 Department of Artificial Intelligence & Software, Kangwon National University, Gangwon-do, Republic of Korea; University of Zaragoza, SPAIN

## Abstract

**Background:**

The norovirus is a major cause of acute gastroenteritis at all ages but particularly has a high chance of affecting children under the age of five. Given that the outbreak of norovirus in Korea is seasonal, it is important to try and predict the start and end of norovirus outbreaks.

**Methods:**

We predicted weekly norovirus warnings using six machine learning algorithms using test data from 2017 to 2018 and training data from 2009 to 2016. In addition, we proposed a novel method for the early detection of norovirus using a calculated norovirus risk index. Further, feature importance was calculated to evaluate the contribution of the estimated weekly norovirus warnings.

**Results:**

The long short-term memory machine learning (LSTM) algorithm proved to be the best algorithm for predicting weekly norovirus warnings, with 97.2% and 92.5% accuracy in the training and test data, respectively. The LSTM algorithm predicted the observed start and end weeks of the early detection of norovirus within a 3-week range.

**Conclusions:**

The results of this study show that early detection can provide important insights for the preparation and control of norovirus outbreaks by the government. Our method provides indicators of high-risk weeks. In particular, last norovirus detection rate, minimum temperature, and day length, play critical roles in estimating weekly norovirus warnings.

## Introduction

The norovirus first emerged in 1968 in Norwalk, Ohio, in the United States of America [1]. The main symptoms of a norovirus infection include diarrhea, vomiting, nausea, and stomach pain. The norovirus can also cause fever, headache, and body aches. A person usually develops symptoms within 12–48 hours after exposure to norovirus. Most people with norovirus illness improve within 1–3 days [2]. The problem with the norovirus is that it can cause multiple infections owing to short-term immunity [3]. In addition, since there are currently no vaccines or specific treatments for the norovirus, the spread of the resultant disease can only be controlled through personal hygiene management and isolation in the event of an outbreak. An outbreak of norovirus infection refers to the case in which two or more norovirus symptoms appear in the same place and within a set period. In the event of an outbreak, patients are restricted from using living facilities for 48 hours after symptoms disappear, and hospitals infected patients in single rooms to prevent contact with other patients [4].

Worldwide, approximately one in every five cases of acute gastroenteritis (inflammation of the stomach or intestines), that leads to diarrhea and vomiting, is caused by norovirus. The norovirus is the most common cause of acute gastroenteritis, with an estimated 685 million cases annually. Approximately 200 million cases are seen among children under five years of age, leading to an estimated 50,000 child deaths every year, mostly in developing countries. However, norovirus infection is a problem in both low- and high-income countries. The norovirus is estimated to cost $60 billion annually worldwide due to healthcare costs and lost productivity [5].

In previous studies, norovirus outbreaks were predicted using machine learning methods [6–8]. These studies generally discuss norovirus outbreaks in view of various environmental variables. Oysters are one of the biggest sources of norovirus, and various studies have predicted oysters to be the source of norovirus outbreaks. Chenar and Deng conducted feature selection using random forest and binary logistic regression to verify the hypothesis, through genetic programming, that oyster norovirus outbreaks are mainly caused only by specific environmental conditions [6]. Similarly, they conducted ANN (Artificial Neural Network) with feature reduction through PCA (Principal Component Analysis) to predict the risk of norovirus outbreaks off the coast of the United States and Mexico [7, 8]. In addition to the machine learning method, the spread of norovirus has been predicted using traditional mathematical modeling [9, 10]. These mathematical models usually have limitations in predicting outbreaks in local areas compared to making predictions about large areas. Tower et al. adopted a new mathematical model that considered the environmental and direct transmission of norovirus, and calculated the reproduction number for environmental and direct transmission on cruise ships [9]. Gaythorpe et al. developed an age-specific mathematical model for norovirus transmission and vaccination using reports of norovirus in Germany [10].

Although Korea has an advanced water supply system, norovirus infections continue to occur. A total of 378 infected individuals were reported in 2005, 3,045 in 2010, and 1,104 in 2015 [11]. An outbreak was also reported during the 2018 Pyeongchang Olympics [12]. The collective norovirus infection in Korea occurred mainly in places of social gatherings, such as schools or nurseries [13]. The risk was greater for people under the age of five years compared to those above the age of five years [14]. Kim and Kim analyzed the correlation between norovirus patients and meteorological characteristics in Korea [13]. Given that the outbreak of norovirus in Korea is seasonal, it is important to predict the start and end of norovirus warnings.

In this study, our objective was to predict weekly warnings and early detection of norovirus in children under the age of five years in South Korea based on meteorological factors. First, we defined weekly norovirus outbreaks and warnings based on the norovirus detection rate. Second, weekly norovirus warnings were estimated using machine learning classification, and the weekly risk index was determined from the classification results. Finally, the start and end weeks of the early detection of norovirus were predicted as changes in the weekly risk index.

## Methods

**Fig 1:**
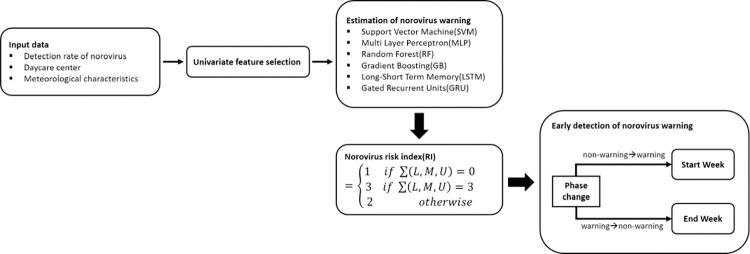
Schematic diagram illustrating how early detection of norovirus was predicted using the classification of machine learning and a risk index. *L*, *M*, and *U* denote the lower, average, and upper bounds of the confidence intervals of the norovirus warning, respectively.

The study approach for the early detection of norovirus using machine learning models is summarized in Fig 1.

First, weekly norovirus outbreak was defined using the weekly norovirus detection rate for 0–5 years old, and the weekly norovirus warning was defined as the presence or absence of a norovirus outbreak within 3 weeks. Second, we estimated norovirus warnings from 2009 to 2018. A classification of machine learning was employed to identify norovirus warnings based on the norovirus detection rate. Third, the norovirus risk index was calculated according to the predicted norovirus warnings in the last three weeks. Additionally, early norovirus detection was predicted using the norovirus risk index. Finally, the performance of the predicted norovirus warning and early norovirus detection was compared for each machine learning method.

### Data collection

**Fig 2:**
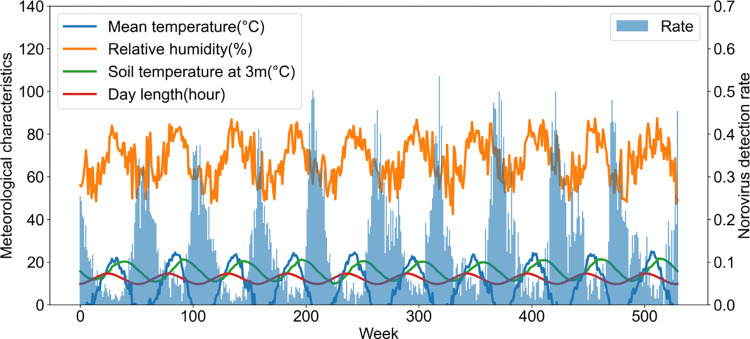
Weekly norovirus detection rate and meteorological characteristics curves (average of temperature, relative humidity, soil temperature at 3m, and day length) from 2009 to 2018 in South Korea.

Our data were categorized according to the detection rate of the norovirus, daycare center, and meteorological characteristics. The weekly detection rate of norovirus in South Korea and the proportion of patients with norovirus among diarrheal patients for a week were collected from the 2009 to 2018 data provided by the KDCA [11]. The number of daycare centers and the population of daycare centers in South Korea were provided by the KOSIS National Statistical Portal [15]. Meteorological data, including the weekly average temperature, maximum temperature, minimum temperature, rainfall, minimum humidity, relative humidity, day length, duration of sunshine, soil temperature at 3 meters, and soil temperature at 5 meters in South Korea, were collected from the 2009 to 2018 data provided by the Korea Meteorological Administration [16]. The norovirus detection rates and meteorological characteristic curves are presented in Fig 2. In addition, we analyzed the publicly available data from previous studies [11, 15, 16]. The datasets used in this study were summarized and anonymized. Therefore, ethical approval was not required for the analysis of anonymized publicly available data.

### Norovirus outbreak and warning

A weekly norovirus outbreak was defined when the detection rate of norovirus for a week was greater than 0.1, as the median value of the weekly norovirus detection rate from 2009 to 2018, as shown in S1 Fig. In addition, categorical—non-warning (0) or warning of norovirus (1)—were based on the occurrence of norovirus outbreaks over the past three weeks; that is, if norovirus outbreaks did not occur over the past three weeks, non-warning is defined; otherwise, norovirus warning is defined.

### Feature selection

To predict weekly norovirus warnings, 14 features (10 meteorological data, 2 care centers’ data, week, and last detection rates) were used. We used the Minmax scaler and feature selection to improve accuracy and reduce computational costs [17]. Among the various feature selections, the univariate feature selection is based on statistical analysis, such as the F-test and chi-squared test [18]. Univariate feature selection was conducted according to the norovirus warning using the F-statistic of the F-test. We selected all features that were defined as statistically significant with a *P*-value < 0.05, except when the correlation coefficient of the correlation analysis was greater than 0.9. Finally, only the variable with the largest F-statistic was selected for the variables with a correlation coefficient of correlation analysis greater than 0.9. The selected important variables were used to estimate norovirus warnings using machine learning classification.

### Estimation of norovirus warning

Weekly norovirus warnings were estimated using the selected features as important variables. We employed six machine learning algorithms for classification:

support vector machine (SVM), multilayer perceptron (MLP), random forest (RF), gradient boosting (GB), long short-term memory (LSTM), and gated recurrent unit (GRU) [19–24].

The SVM algorithm is expressed as a boundary in the space where data exists, and it determines the boundary with the largest width. It can be used not only in linear but also in nonlinear classification [19]. The type of kernel used in the algorithm was a radial basis function.

The MLP algorithm with perceptrons has the input layers, the output layers, and several stacked hidden layers that are advantageous in solving nonlinear classification problems [20]. We used 100 hidden layers, Relu activation function, and adaptive moment estimation (Adam) optimizer in the MLP algorithm.

The RF algorithm introduces additional randomness each time a tree is constructed using a bagging method, with each tree having a different characteristic induced by randomness, which results in decorrelations in the predictions of each tree [21]. The number of trees used in the RF algorithm was 20, the number of maximum depth and leaf node were 10 and 5, respectively.

The GB algorithm is an ensemble of weak prediction models, which are decision trees. It does not use randomness but sequentially generates trees using a method of supplementing the errors of previous trees, and uses gradient descent to compensate for errors [22]. The number of trees, maximum depth, and leaf nodes for trees used in the GB algorithm were 20, 10, and 5, respectively, which were the same values used for the RF algorithm.

The LSTM algorithm is an artificial recurrent neural network (RNN) architecture, a machine learning algorithm, and a suitable gradient-based method. LSTM comprises three gates—forget gate, input gate, and output gate [23]. We built 30-layers, Relu activation function, and Adam optimizer in the LSTM algorithm.

The GRU algorithm uses each recurrent unit to adaptively capture dependencies of different time scales. GRU is similar to LSTM, but it is much simpler to compute and implement [24] compared with LSTM. We bulit 50-layers, Relu activation function, and Adam optimizer in the GRU algorithm.

Data of selected features and warnings were used as training data from 2009 to 2016 and testing data from 2017 to 2018. The training data had 424 cases, which includes 209 (49.29%) positive cases (warning of norovirus); and the testing data had 106 cases, which includes 64 (60.38%) positive cases. The prevalence rates of training and test cases were not significantly different. Subsequently, the average and 95% confidence intervals (95% CIs) for 1000 simulation results of the training and test data were obtained. To evaluate the performance of machine learning, the three measurements of accuracy, F1-score, and area under the ROC curve (AUC) were compared. Python language version 3.9.7 with TensorFlow 2.6.0 and scikit-learn 1.0.1 was used. In addition, svm.SVC, MLPClassifier, RandomForestClassifier, and GradientBoostingClassifier functions of scikit-learn, LSTM and GRU functions of TensorFlow were used to simulate 6 classification algorithms.

### Norovirus risk index

The average and 95% confidence interval for a total of 1000 simulation results for the classification of machine learning algorithms were obtained, where M, U, and L denote the average, upper, and lower bounds of the confidence intervals of the norovirus warning, respectively. Consequently, the weekly risk index was defined as safety (1), caution (2), and danger (3) according to the weekly combination of (L, M, U) simulation results and 1000 simulations of train and test data, as follows:

$$\mathrm{Risk}\;\mathrm{index}\;(\mathrm{RI})=\left\{ \begin{array}{l} 1\;if\;\sum \left(L,M,U\right)=0\\ 3\;if\;\sum \left(L,M,U\right)=3\\ \;2\;\mathrm{otherwise} \end{array}\right.$$

For example, if weekly L, M, and U are 0 (non-warning), 1 (warning), and 1, ∑(*L,M,U*) is 2, then RI is calculated as 2 (caution).

### Norovirus early detection

We defined the warning and non-warning phases as the weekly time interval in which the weekly norovirus warning persisted and the week in which the non-warning persisted, respectively. In addition, we defined the start and end of the early detection weeks as the weeks in which the warning phase begins in the non-warning phase and the weeks in which the non-warning phase begins in the warning phase. At that time, we predicted the start and end week of early detection as a week time interval in which the risk index changed from 1 to 3 and a week time interval in which the risk index changed from 3 to 1.

## Results

### Feature selection

**Fig 3:**
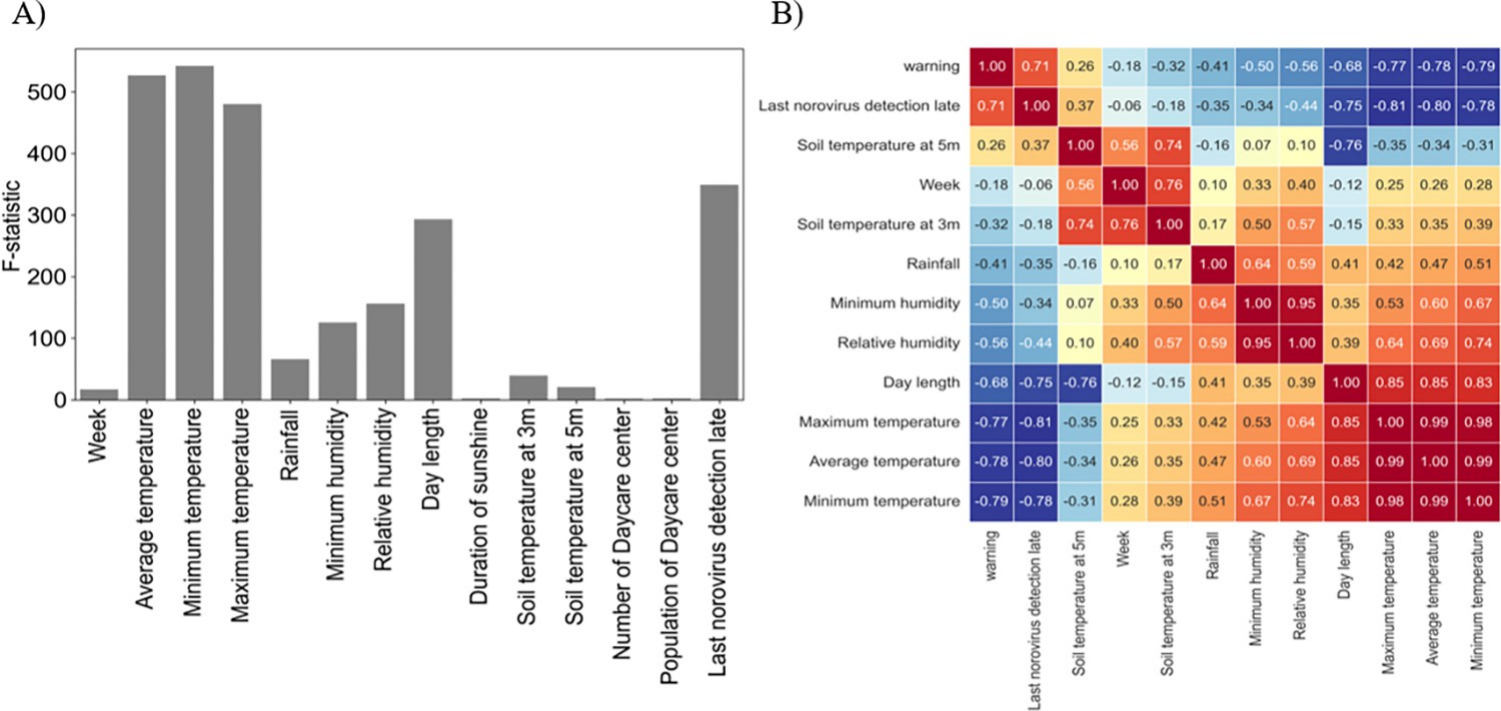
Results of feature selection. **A**) The F-statistic for 14 features computed from the univariate feature selections about weekly norovirus warning. **B**) Correlation analysis between the weekly norovirus warning and 11 selected features from the univariate feature selections.

The results of univariate feature selection for weekly norovirus warnings are shown in S1 Table and Fig 3A. We selected 11 important features with a *P*-value < 0.05, which included week, weekly average of temperature, maximum temperature, minimum temperature, rainfall, minimum humidity, relative humidity, day length, soil temperature at 3 meters, soil temperature at 5 meters and last norovirus detection rate. Meals that are provided in daycare centers are known to be one of the sources of large norovirus outbreaks [25]; however, the number of daycare centers and the population of daycare centers were not selected as important features in the univariate feature selection. Correlation analysis was performed between the 11 selected features and weekly norovirus warnings (Fig 3B). There was a positive correlation between weekly norovirus warning, last norovirus detection rate, and soil temperature at 5 m with a correlation coefficient of 0.71 and 0.26 and a negative correlation of weekly norovirus warning with the three features of temperature (−0.79 –−0.77), day length (−0.68), the two features of humidity (−0.56 –−0.50), rainfall (−0.41), soil temperature at 5 m (−0.32), and week (−0.18). Moreover, we observed that the three features of temperature and the two features of humidity had a correlation coefficient than 0.9. The weekly minimum temperature and relative humidity for the three features of temperature and the two features of humidity were included as important features because they showed the highest score in the univariate feature selection (S1 Table). Finally, we obtained eight important features to estimate norovirus warning using machine learning classification: week, weekly minimum temperature, rainfall, relative humidity, day length, soil temperature at 3 m, soil temperature at 5 m and the last norovirus detection rate.

### Estimation of norovirus warning

**Fig 4:**
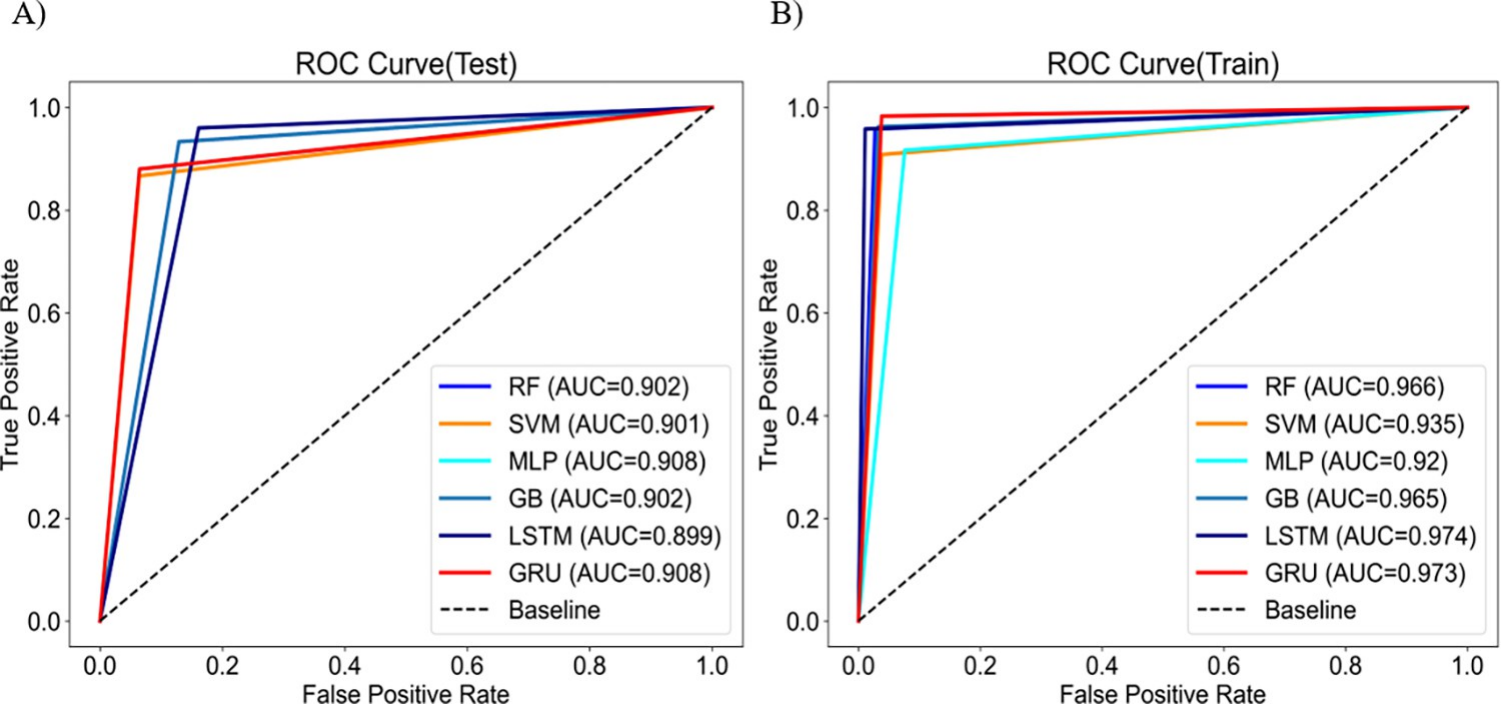
ROC curve comparing 6 machine learning algorithms for **A**) train and **B**) test data.

Eight important features were used to predict weekly norovirus warnings for training data from 2009 to 2016 and test data from 2017 to 2018 by employing six machine learning algorithms. Table 1 shows the performance with the average accuracy, F1-score, and AUC of weekly norovirus warning prediction for 1000 simulations of training and test data. The accuracy and F1-score of the training data was found to be than 90%, while those of the test data were in the range of 88–95%. The LSTM algorithm exhibited higher accuracies of 97.2 and 92.5% for the training and test data, respectively. In addition, Fig 4 shows the AUC results from the six machine learning algorithms. Given the AUC results, the LSTM algorithm showed the highest value of 0.974 for the training data, however, the AUC for the test data was generally similar at 0.899–0.908. This indicates that LSTM is the most suitable algorithm for predicting weekly norovirus warnings.

**Table 1:** Performance of train and test data for predicting weekly norovirus warning, using the 6 machine learning algorithms.

Estimator	Training cases (*n* = 424)			Test cases (*n* = 106)		
	Accuracy	F1-score	AUC	Accuracy	F1-score	AUC
SVM	0.931	0.938	0.935	0.887	0.915	0.901
MLP	0.92	0.928	0.92	0.896	0.923	0.908
RF	0.965	0.968	0.966	0.915	0.94	0.902
GB	0.965	0.969	0.965	0.915	0.94	0.902
LSTM	0.972	0.974	0.974	0.925	0.947	0.899
GRU	0.974	0.978	0.974	0.9	0.923	0.899

### Norovirus early detection

We estimated the weekly risk index of norovirus based on the average and 95% confidence interval of the prediction of weekly norovirus warnings for 1000 simulations of train and test data. In addition, we predicted the start and end weeks of early detection as a week time interval in which the risk index changed from 1 to 3 and a week time interval in which the risk index changed from 3 to 1.

**Fig 5:**
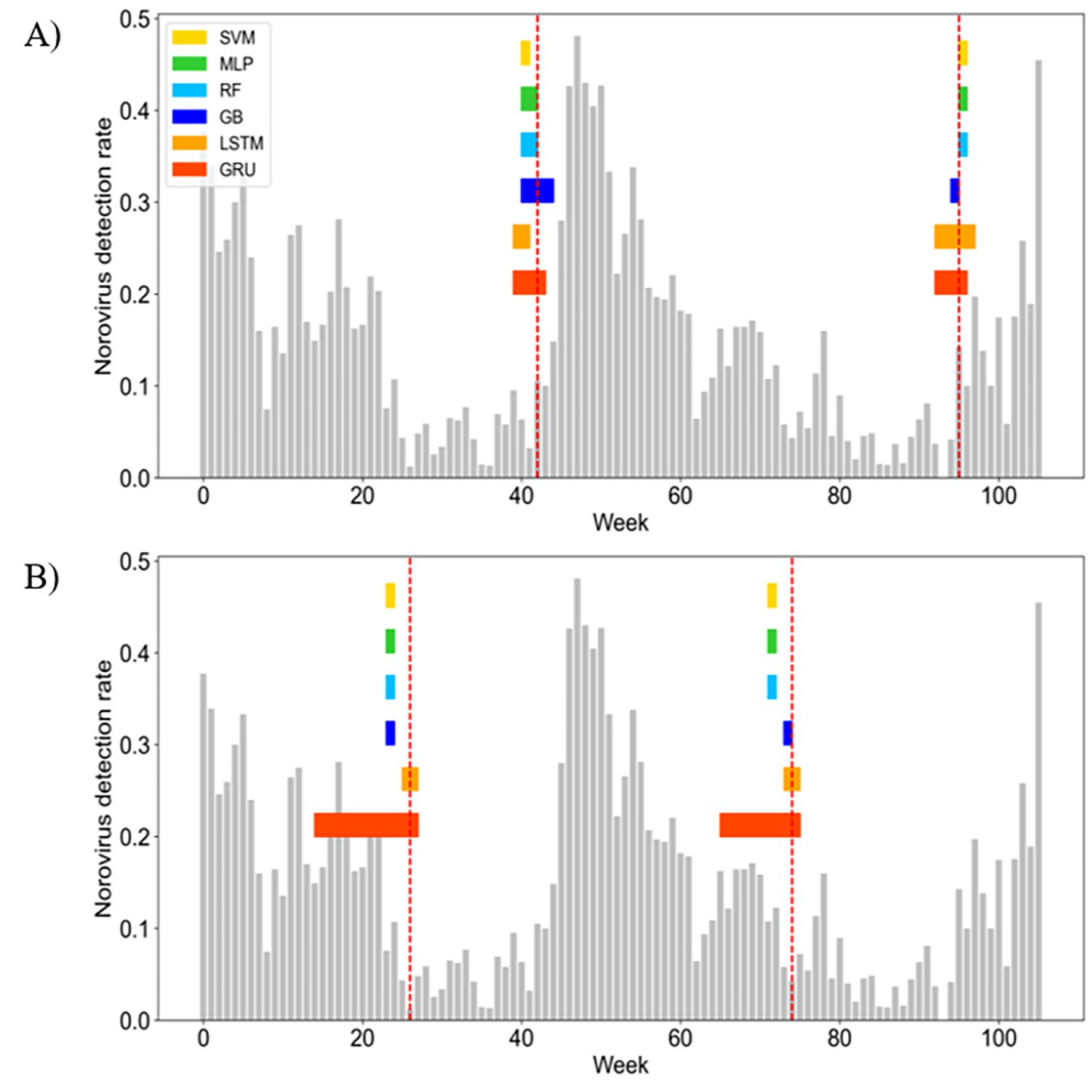
Comparison of early detection of norovirus between six machine learning algorithms for test data. A) start week of early detection and B) end week of early detection. The vertical red dotted lines indicate an observed start and end week of norovirus warning phase. The horizontal color bars indicate a predicted interval for start and end week of norovirus warning phase using SVM (yellow), MLP (green), RF (pale blue), GB (blue), LSTM (orange), and GRU (red). The gray bars indicate an observed weekly detection rate of norovirus.

S2 Fig and Fig 5 show the predicted interval of early detection of norovirus using six machine learning algorithms for train and test data, respectively. The observed early detection week of the norovirus was significantly included in the predicted interval of early detection in LSTM and GRU. Table 2 shows that the observed start and end week of early detection prediction of the norovirus was substantially accurate in the LSTM and GRU algorithms. However, in the case of the GRU algorithm, the prediction interval of early detection is too long. Only the LSTM algorithm accurately predicted the observed start and end week of early detection of the norovirus within a 3-week range for both training and test data.

**Table 2:** Comparison of observed start and end week of norovirus warning phase and predicted start and end week time interval of early detection of norovirus in 6 machine learning algorithms.

**Year**	**Observed start week**	**Prediction (train)**						**Observed end week**	**Prediction (train)**					
		**SVM**	**MLP**	**RF**	**GB**	**LSTM**	**GRU**		**SVM**	**MLP**	**RF**	**GB**	**LSTM**	**GRU**
**2009**	42	43	43	43	42~44	41~42	40~42	12	15	16~18	16~18	14	12~13	13~18
**2010**	40	42	42~43	42~43	42	40~41	39~41	17	18	18~20	18~20	18	16~17	13~18
**2011**	43	44	44	44	42~44	41~43	39~43	15	17	18~19	18~19	17~19	15~16	14~17
**2012**	40	42	42	42	42	40~41	39~41	16	17	17~19	17~19	16~17	13~17	13~17
**2013**	40	43	43	43	42	40~41	40~41	10	13	16~17	16~17	12	10~11	10~15
**2014**	40	43	43	43	42~43	40~41	39~41	23	21	21	21	24	23~24	18~23
**2015**	40	43	41~43	41~43	42	39~41	40~41	13	14	17~18	17~18	14~15	13	13~23
**2016**	40	42	42~43	42~44	42	40~41	40~42	19	16	16	16	16	19	14~23
**year**	**observed start week**	**prediction (test)**						**observed end week**	**prediction (test)**					
		**SVM**	**MLP**	**RF**	**GB**	**LSTM**	**GRU**		**SVM**	**MLP**	**RF**	**GB**	**LSTM**	**GRU**
**2017**	43	41	41~42	41~42	41	40~41	40~44	27	24	24	24	24	26~27	15~27
**2018**	43	43	43	43	42	40~44	40~44	22	19	19	19	21	21~22	13~22

**Fig 6:**
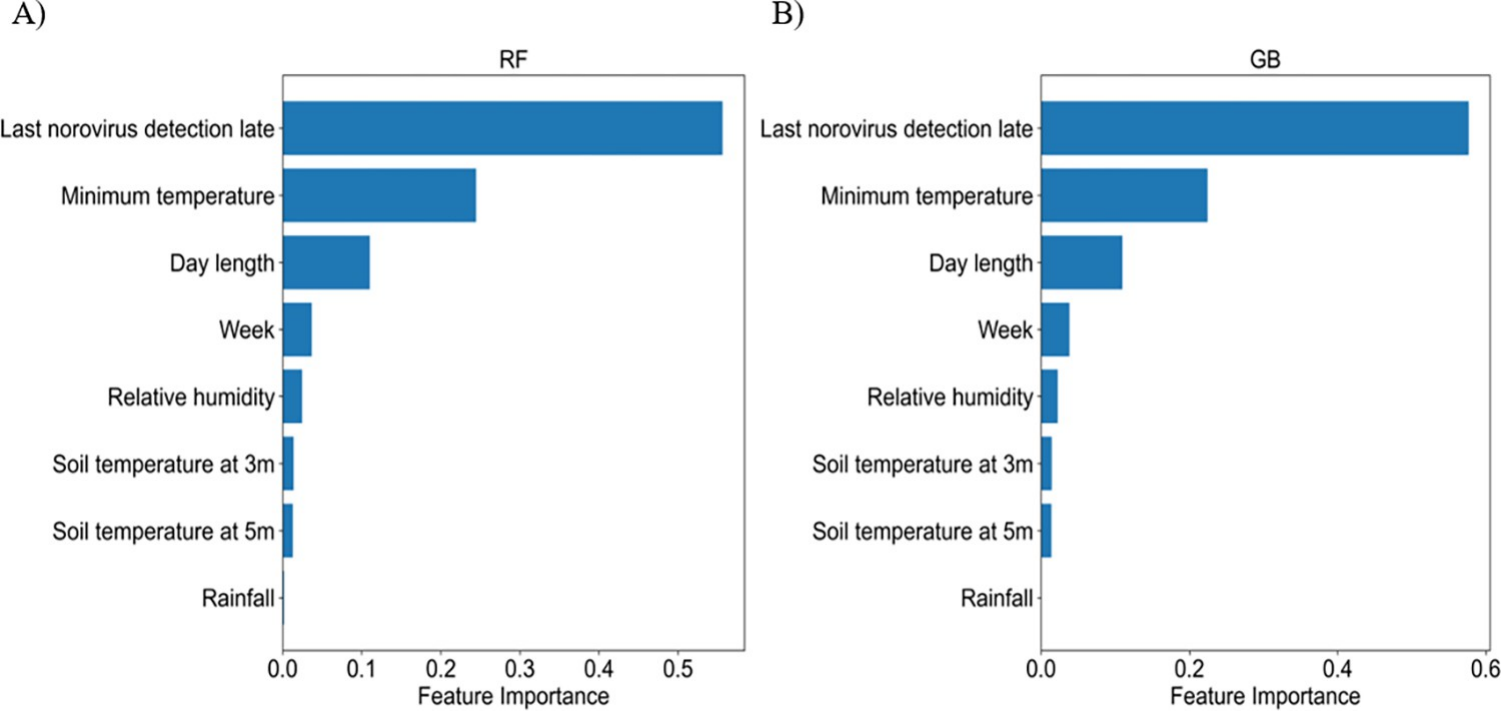
Feature importance of **A**) RF and **B**) GB.

Although the best machine learning method in our study is the LSTM, feature importance was calculated through RF and GB which are capable of analyzing feature importance. Fig 6 shows that the last norovirus detection rate was found to be the most important feature for RF and GB (0.55, 0.58). The feature importance of the minimum temperature (0.25, 0.23) and day length (0.11, 0.11) were higher than 0.1 for both RF and GB, respectively. Meteorological factors with high F-statistics in univariate feature selection showed higher feature importance.

## Discussion

This study elucidated the weekly warning characteristics of norovirus infection in South Korea, and explored possible meteorological factors related thereto. Our study aims at predicting weekly warnings and early detection of the norovirus in children under the age of five years in South Korea. We used six machine-learning algorithms and calculated the risk index to achieve these two objectives. Accordingly, the results of our study can be summarized as follows:

First, eight important features were selected to estimate norovirus warnings using univariate feature selection and correlation analysis, including week, weekly minimum temperature, rainfall, relative humidity, day length, soil temperature at 3 m, soil temperature at 5 m and the last norovirus detection rate (Fig 3). Second, we predicted weekly norovirus warnings using six machine learning algorithms for test data from 2017 to 2018 using training data from 2009 to 2016. The weekly norovirus non-warning was defined if weekly norovirus outbreak did not occur over the past three weeks; otherwise, norovirus warning was defined. As a result, LSTM was the best algorithm, with 97.2% and 92.5% accuracies in the training and test data, respectively as shown in Table 1. Third, we proposed a novel method in which early norovirus detection was predicted using the calculated norovirus risk index. The weekly risk index of norovirus was estimated to be the average and 95% confidence interval of the prediction of weekly norovirus warning. As shown in Fig 5, the interval of early detection of norovirus from 2017 to 2018 was calculated using six machine-learning algorithms based on the estimated weekly norovirus warning. Table 2 shows that the LSTM algorithm accurately predicted the observed start and end weeks of early detection of norovirus within a 3-week range for both the training and test data. Finally, we analyzed the feature importance using the RF and GB algorithms. The feature importance of minimum temperature (0.25, 0.23) and day length (0.11, 0.11) in meteorological factors was higher than 0.1 for both RF and GB, respectively.

We proposed a novel method for the early detection of norovirus using the calculated norovirus risk index. To the best of our knowledge, this is the first study to use machine learning algorithms to predict early detection in children under the age of five based on meteorological factors. In particular, the LSTM algorithm showed high accuracy in predicting warnings and the early detection of norovirus.

Our study has several limitations. First, the data on norovirus detection rate are sample data only from some hospitals in South Korea, and does not represent Korea as a whole. Next, because the meteorological factors used were the average weekly values of Korea as a whole, it is difficult to reflect regional weather changes in the prediction. However, in Korea, there is little regional variation in the meteorological factors. Finally, it is difficult to predict norovirus outbreaks that eventually occur in spring [25]. However, this was not a problem in predicting the overall trend of warning and early detection of norovirus, which were the goals of our study.

## Conclusions

Our study predicted weekly warnings and early detection of the norovirus in children under the age of five years in South Korea using meteorological factors. The results of early detection provide important insights for the preparation and control of norovirus outbreaks by the government. Our method provides indicators of high-risk weeks. In particular, the last norovirus detection rate, minimum temperature, and day length play critical roles in estimating weekly norovirus warnings. To prevent norovirus outbreaks, our findings emphasize the need to implement norovirus risk alerts based on weekly weather forecasts. In addition, this study was the first to propose a method for predicting early detection in children under the age of five years in South Korea and our findings indicated that high accuracy prediction, by applying machine learning algorithms, is feasible.

## Supporting information

S1 Table Univariate feature selection to predict norovirus warning. (DOCX)

S1 Fig Histogram of weekly norovirus detection rate, the blue dotted line represents the Q1 value, the red dotted line is the Q2 value, and the green dotted line is the Q3 value. (TIF)

S2 Fig Comparison of early detection of norovirus between six machine learning algorithms of training data. A) Start week of early detection; B) End week of early detection. The vertical red dotted lines indicate the observed start and end week of norovirus warning phase. The horizontal color bars indicate the predicted intervals for start and end week of norovirus warning phase using SVM (yellow), MLP (green), RF (pale blue), GB (blue), LSTM (orange), and GRU (red). The gray bars indicate the observed weekly detection rate of norovirus.
(TIF)

## Abbreviations

LSTM: long short-term memory
PCA: principal component analysis
SVM: support vector machine
RF: random forest
GB: gradient boosting
MLP: multilayer perceptron
GRU: gated recurrent unit
CI: confidence interval
