# Supplementary material for: Early detection of norovirus outbreak using machine learning methods in South Korea
Source: PLoS One. 2022 Nov 16;17(11):e0277671. doi: 10.1371/journal.pone.0277671 (PMC9668130; doi:10.1371/journal.pone.0277671)
Supplement: S1 Table — (DOCX) [file pone.0277671.s001.docx]

**Table S1.** Univariate feature selection to predict norovirus warning

| Features | Occurrence | |
| --- | --- | --- |
|  | F-value | P-value |
| Week | 17.14 | <0.01 |
| Average temperature | 526.71 | <0.01 |
| Minimum temperature | 542.55 | <0.01 |
| Maximum temperature | 480.33 | <0.01 |
| Rainfall | 66.144 | <0.01 |
| Minimum humidity | 125.65 | <0.01 |
| Relative humidity | 156.17 | <0.01 |
| Day length | 293.52 | <0.01 |
| Duration of sunshine | 2.47 | 0.12 |
| Soil temperature at 3m | 39.64 | <0.01 |
| Soil temperature at 5m | 20.93 | <0.01 |
| Number of daycare center | 1.88 | 0.17 |
| Population of daycare center | 2.46 | 0.12 |
| Last norovirus detection late | 349.21 | <0.01 |
