# Supplementary figures and images for: Early detection of norovirus outbreak using machine learning methods in South Korea
Source: PLoS One. 2022 Nov 16;17(11):e0277671. doi: 10.1371/journal.pone.0277671 (PMC9668130; doi:10.1371/journal.pone.0277671)

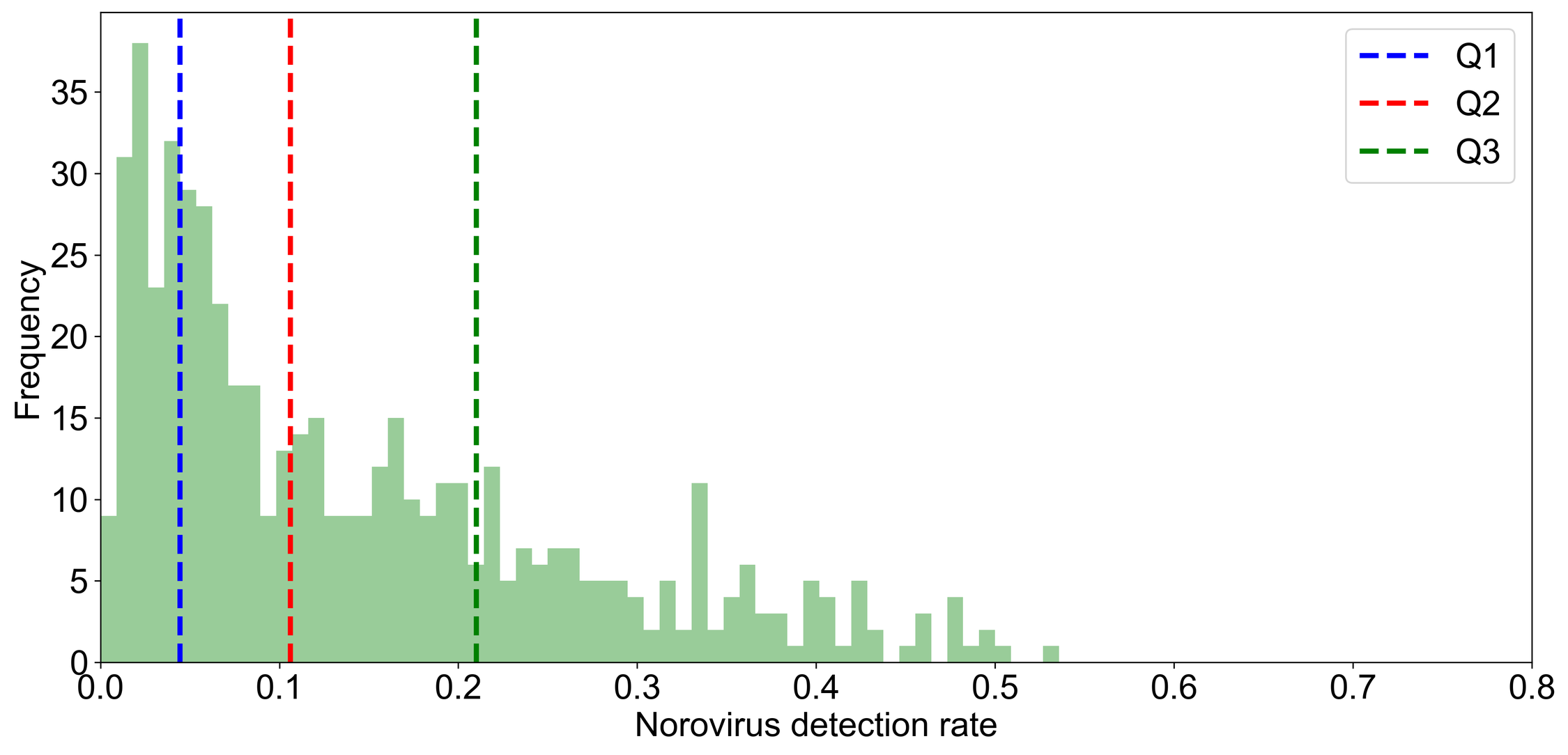

Supplement: S1 Fig — (TIF) [file pone.0277671.s002.tif]

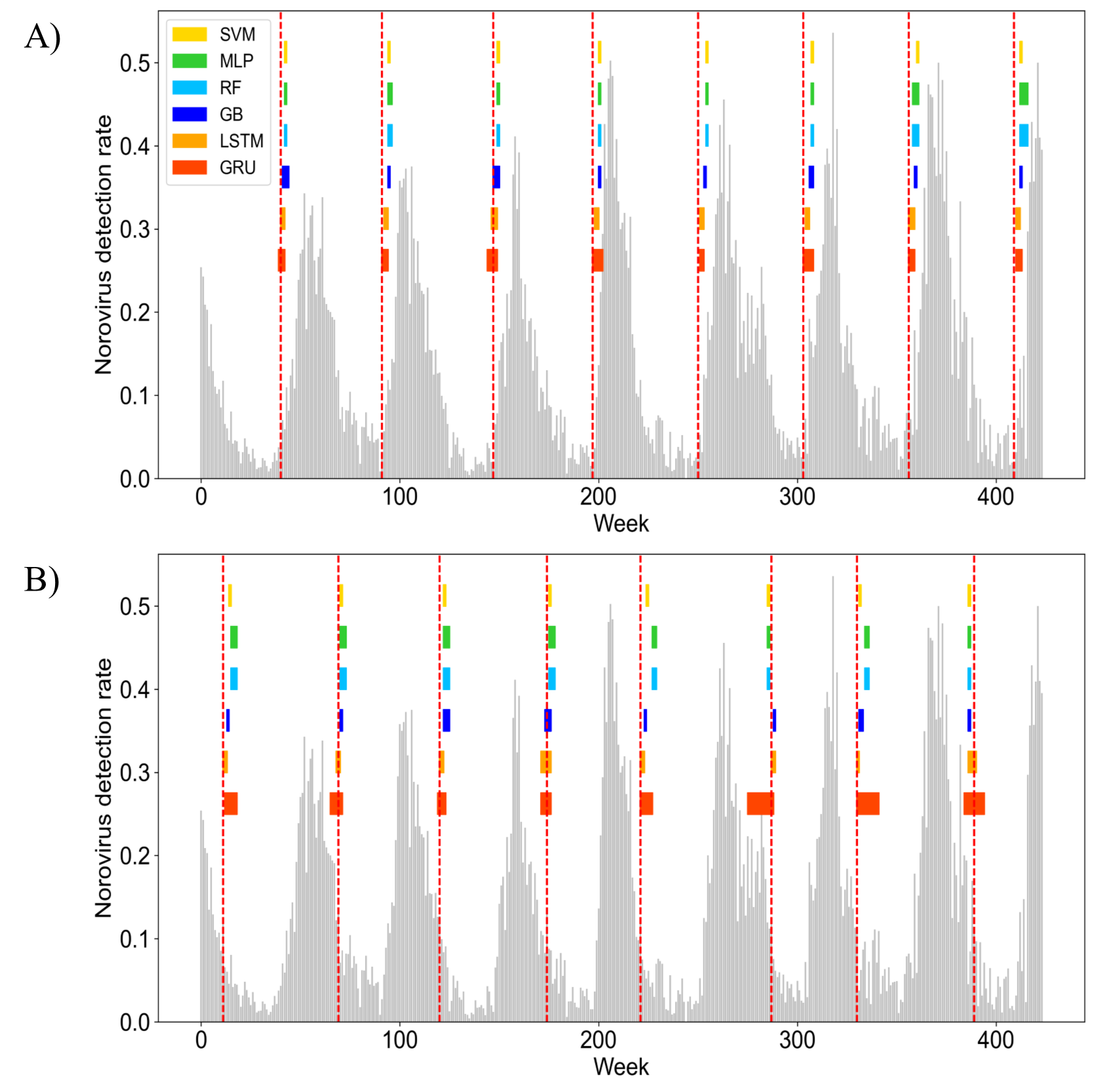

Supplement: S2 Fig — A) Start week of early detection; B) End week of early detection. The vertical red dotted lines indicate the observed start and end week of norovirus warning phase. The horizontal color bars indicate the predicted intervals for start and end week of norovirus warning phase using SVM (yellow), MLP (green), RF (pale blue), GB (blue), LSTM (orange), and GRU (red). The gray bars indicate the observed weekly detection rate of norovirus. (TIF) [file pone.0277671.s003.tif]
